# Pulmonary ultrasound-guided management of delayed pulmonary edema secondary to nitric acid fumes inhalation: a case report

**DOI:** 10.1186/s12890-025-03758-y

**Published:** 2025-07-02

**Authors:** Zhenqing Zhao, Yinbing Jin, Huaibao Ma, Mingfeng Lu

**Affiliations:** 1 Department of Emergency Department, Northern Jiangsu People’s Hospital Affiliated to Yangzhou University, Yangzhou, 225001 China; 2 Xuzhou Medical University, Xuzhou, 221000 China

**Keywords:** Nitric acid fumes, Pulmonary edema, Pulmonary ultrasound

## Abstract

**Background:**

Concentrated nitric acid can form nitrogen oxides through a spontaneous redox reaction. After inhalation, it has a stimulating effect on the respiratory tract and can cause inhalation lung injury. This lung injury is mainly manifested as non-cardiogenic pulmonary edema in imaging, and pulmonary ultrasound can dynamically monitor the severity of pulmonary edema at the patient’s bedside.

**Case presentation:**

This article describes a 52-year-old female case who experienced chest tightness and dyspnea 8 h after inhaling nitric acid fumes and was sent to the emergency room. Chest CT suggested diffuse exudation in both lungs, and dynamic B-lines could be seen by bedside pulmonary ultrasound. During the treatment of this patient, we adjusted the dosage of hormones and the oxygen therapy plan in a timely manner according to the evolution of B-lines under ultrasound. After treatment, her clinical symptoms gradually improved, and the re-examination of chest CT showed that the exudation in both lungs was gradually absorbed.

**Conclusion:**

This case reminds clinicians to be alert to the occurrence of delayed pulmonary edema when treating patients who inhale nitric acid fumes. At the same time, dynamic examination of bedside pulmonary ultrasound is of great value in the treatment of such patients.

**Supplementary Information:**

The online version contains supplementary material available at 10.1186/s12890-025-03758-y.

## Introduction

Nitric acid is commonly used as a cleaner in etching and industrial manufacturing. As a strong oxidizing inorganic acid, its high volatility and corrosiveness have various pathological damages to human respiratory tract [1]. After inhaling nitric acid fumes, the clinical process shows a biphasic feature: the symptoms of the upper respiratory tract, such as irritating cough and sore throat, are the main manifestations within 0 to 4 h after exposure; after 4 to 6 h, progressive dyspnea and hypoxemia occur, and in severe cases, it can rapidly evolve into ARDS [2].

Imaging techniques are of great value in the clinical assessment of non-cardiogenic pulmonary edema caused by inhalation of irritating gases. Common methods include chest X-ray, CT, and pulmonary ultrasound. This article reports a case of the treatment of delayed pulmonary edema that occurred 8 h after occupational exposure to nitric acid fumes. When the patient visited the hospital, she presented with acute pulmonary edema symptoms, and bedside pulmonary ultrasound showed diffuse B-line signs in both lungs; after treatment, as the condition relieved, the distribution range of B-lines gradually decreased. It is worth noting that the application of bedside ultrasound in such poisoning cases is rarely mentioned in the previous literature. The retrospective analysis of this case not only deepens the understanding of the clinical characteristics of nitric acid fume poisoning but also shows the value of bedside pulmonary ultrasound as an auxiliary tool in the dynamic monitoring of the evolution of pulmonary edema, providing a visual basis for the optimization of drug treatment regimens and the evaluation of prognosis.

## Case presentation

The 52-year-old female patient is a chemical plant worker. According to the patient’s description, she accidentally inhaled nitric acid fumes at work. At first, she only had a mild cough. About 8 h later, she developed chest tightness, shortness of breath, expectoration, and the sputum was yellow foamy, accompanied by nausea, so she went to the emergency room. Physical examination at the time of admission: pulse 90 beats/min, respiratory rate 22 breaths/min, blood pressure 188/99 mmHg, and peripheral oxygen saturation 97% (nasal cannula 5 L/min). At this time, the patient’s P/F ratio was measured at 277 mmHg. The patient was conscious, the pharyngeal mucosa was not edematous, and the lips were not cyanotic. Auscultation revealed symmetrical breathing sounds in both lungs, and moist rales could be heard. The admission diagnosis was inhalation of toxic gas pneumonia and hypertension.

**Fig. 1:**
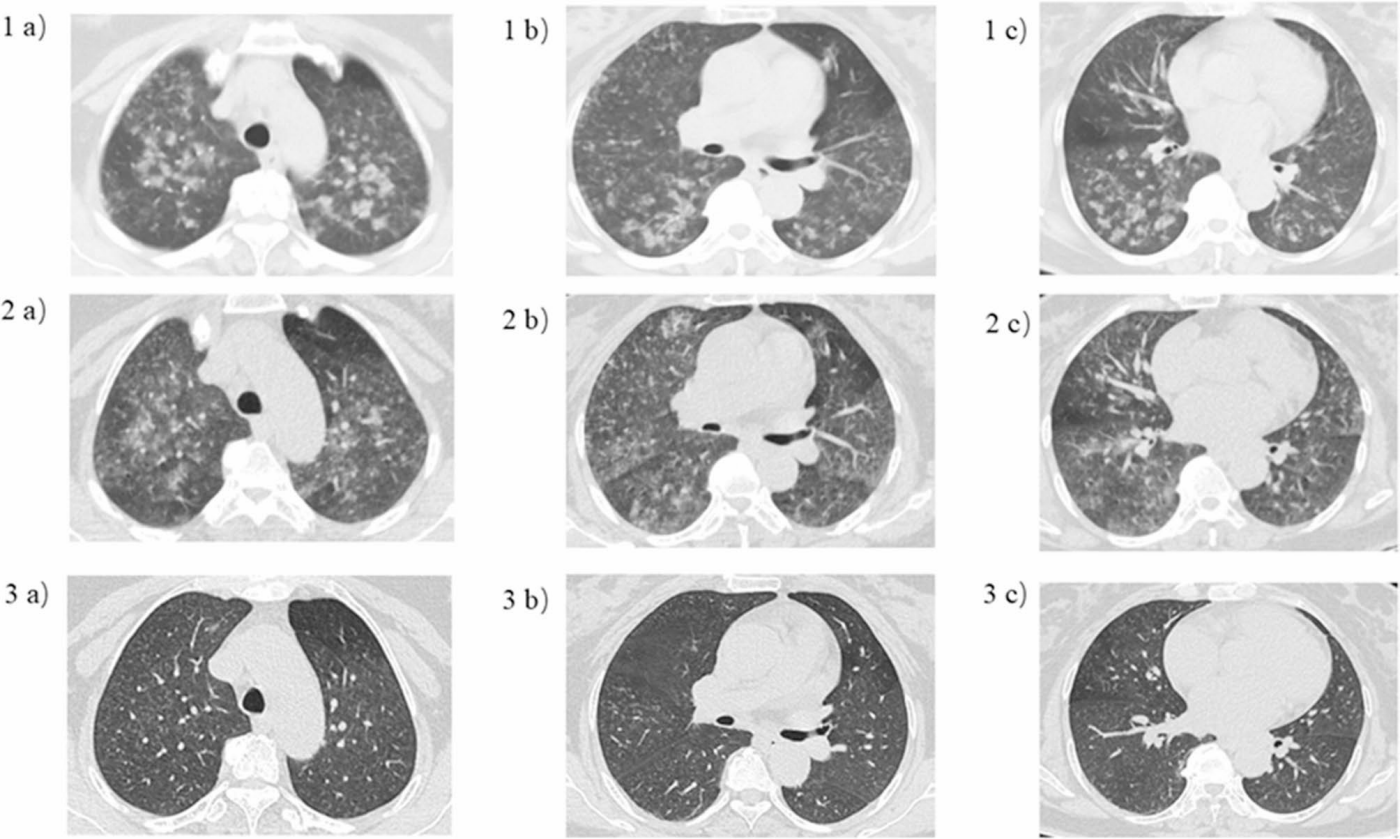
Comparison of chest CT with the same plain layer at different times. (**1a**-**1c**) Chest CT 8 h after inhaling nitric acid fumes: Diffuse exudation can be seen in upper, middle and lower lungs. (**2a**-**2c**) After inhaling for 3 days: Diffuse exudation is similar to before. (**3a**-**3c**) After inhaling for 5 days: the exudation is obviously absorbed than before

**Fig. 2:**
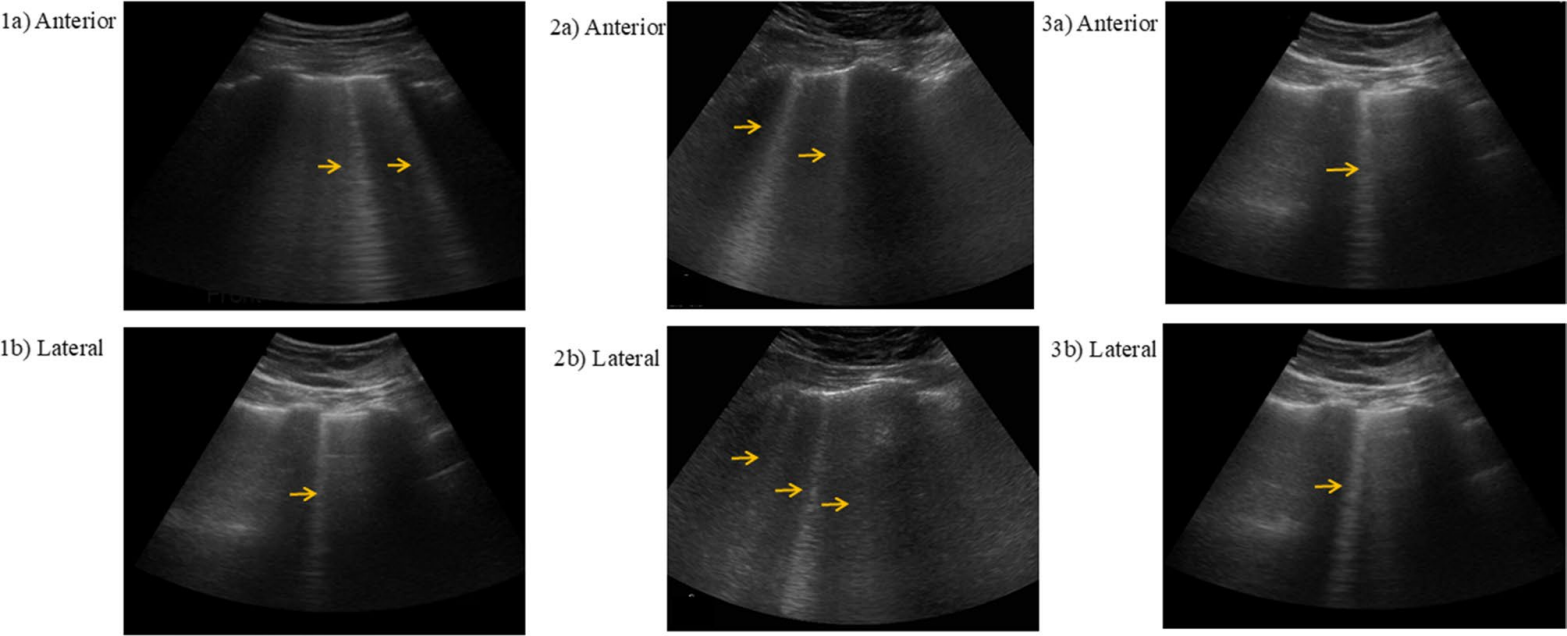
Lung ultrasound at different time after inhaling nitric acid fumes (The arrows refer to B-lines). (**1a**, **1b**) Lung ultrasound 8 h after inhaling nitric acid fumes: Clear laser-like straight lines with “comet tail” characteristics, that is, B-lines, can be seen in the anterior and lateral chest (All three). (**2a**, **2b**) After inhaling for 3 days: The number of B-lines has increased significantly compared with before (All five). (**3a**, **3b**) After inhaling for 5 days: The number of B-lines is less than before (All two)

**Fig. 3:**
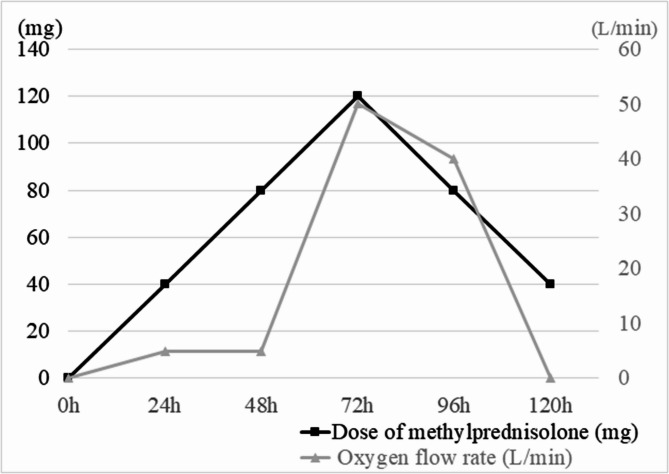
Dose of methylprednisolone and oxygen flow rate at different times after inhaling nitric acid fumes. The black line represents the dose of methylprednisolone, and the gray line represents the oxygen flow rate, in which HFNC is given 72 h after inhalation

After the patient entered the emergency room, the therapeutic regimen comprised oxygen therapy, aminophylline for bronchodilation, and adjunctive budesonide nebulization. Given the patient’s presentation with yellow frothy sputum and abnormal pulmonary findings, empirical cefotaxime sodium administration was initiated due to concerns regarding secondary bacterial infection. Chest CT suggested diffuse exudation in both lungs (Fig. 1). At the same time, referring to the research of the pulmonary ultrasound scoring system, the patient’s anterior and lateral chest was examined by pulmonary ultrasound, and a total of 3 B-lines could be seen (Fig. 2), and 40 mg of methylprednisolone was intravenously injected. On the third day, the patient complained of aggravated chest tightness and asthma. The lowest pulse oxygen saturation under nasal cannula oxygen inhalation dropped to 82%, and the respiratory rate increased to 30 breaths/min. The lung moist rales were louder than before. At this time, chest CT showed that the exudation of both lungs was similar to that before (Fig. 1). But pulmonary ultrasound examination was performed again, a total of 5 B-lines were seen in the patient’s anterior and lateral chest, which was more than before (Fig. 2). Furthermore, P/F ratio declined to 105 mmHg. Bedside echocardiography demonstrated a left ventricular ejection fraction (LVEF) of 65% with normal segmental wall motion. The serum NT-proBNP level was 164 pg/mL (normal range: 20–300 pg/mL). No clinical signs of volume overload were observed, such as jugular venous distension or peripheral edema. These findings collectively indicated worsening non-cardiogenic pulmonary edema in the patient. Therefore, a total of 120 mg of methylprednisolone was intravenously injected to the patient on the same day. At the same time, to improve hypoxemia, nasal high-flow oxygen therapy was given, with the inhaled oxygen flow rate of 50 L/min and the inhaled oxygen concentration of 30%. After treatment, the patient’s dyspnea gradually alleviated. On the fifth day, the cefotaxime sodium was discontinued following clinical improvement and negative sputum culture results. The re-examination of chest CT suggested that the exudation in both lungs was significantly absorbed compared with before (Fig. 1), and the moist rales in both lungs disappeared on auscultation. At this time, the pulmonary ultrasound examination was re-examined and a total of 2 B-lines could be seen in the anterior and lateral chest (Fig. 2), P/F ratio was measured at 572 mmHg. so the high-flow support conditions were gradually reduced until they were discontinued. Oral prednisolone (40 mg/d) was started on the fifth day of treatment, and then the dose was gradually reduced (5 mg per week) (Fig. 3).

Half a year later, the patient was followed up. The patient said that she was still working in the chemical plant. At present, the chest tightness and asthma have improved compared with before, which does not affect normal life and physical labor, and only occasionally feels chest tightness at night.

## Discussion

The volatile fumes of nitric acid can cause serious and extensive damage to the body’s mucous membranes. The clinical manifestations can range from mild irritation of the upper respiratory tracts to third-degree burns of the respiratory tracts. When high-concentration nitric acid fumes reach the patient’s lower respiratory tract, it can cause inhalation lung injuries such as pulmonary edema and obliterative bronchitis [3]. Acute non-cardiogenic pulmonary edema and pulmonary hemorrhage are the most fatal complications of nitric acid inhalation [4, 5]. For such patients, accurate differentiation between cardiogenic and non-cardiogenic pulmonary edema is crucial. In this case, echocardiography demonstrated a preserved LVEF of 65% during treatment, with normal NT-pro BNP levels and absence of clinical signs indicative of volume overload. These characteristic findings definitively support the diagnosis of nitrate fume-induced non-cardiogenic pulmonary edema.

Acute exposure leads to immediate chest pain, wheezing, cough, palpitations, nausea and vomiting, and sweating; some patients will have delayed symptoms, and this delayed reaction has been described in most reports of severe nitric acid fume inhalation injury, usually occurring 4 to 24 h after inhalation of nitric acid fumes. For example, our patient developed gradually aggravated pulmonary edema symptoms 8 h after inhaling nitric acid fumes, manifested as dyspnea and hypoxemia. Therefore, when patients are exposed to nitric acid fumes, the possibility of delayed respiratory tract damage should be considered. There are reports that severe delayed pulmonary edema can lead to the death of patients. For example, Murphy et al. reported a case of death caused by non-cardiogenic pulmonary edema after acute occupational nitric acid exposure [1]. Therefore, early identification of potential disease changes and giving reasonable treatment are crucial for saving lives.

At present, the reports of nitric acid fume inhalation poisoning are mainly case reports, and these reports have proposed some treatment plans, mainly including respiratory support, glucocorticoids, and inhaled bronchodilators. Although evidence universally supporting prophylactic antibiotics in pure inhalation injury remains limited, certain clinical case reports have documented antibiotic use in infection-associated inhalational lung injury [6–8]. In the present case, the emergence of yellow frothy sputum coupled with chest CT findings demonstrating scattered diffuse pulmonary infiltrates raised substantial concern for infection, prompting empirical antibiotic therapy. Subsequent microbiological testing failed to confirm infection, leading to prompt antibiotic discontinuation.

Glucocorticoids may exert beneficial effects in controlling nitrate fume-induced lung injury, particularly when administered during the early clinical phase [9, 10]. Furthermore, the study by Zhang et al. demonstrated that glucocorticoids are significantly effective in treating acute irritant gas-induced pulmonary edema, with their potential mechanism of action being the improvement of capillary permeability and reduction of exudation [11]. For such cases, it is often necessary to use intravenous and inhaled glucocorticoids in combination. In this case, the patient was promptly given intravenous and inhaled glucocorticoids during the hospital stay, and the dosage of hormones was adjusted in a timely manner according to the severity of non-cardiogenic pulmonary edema. The patient’s pulmonary edema was rapidly relieved. After discharge, oral glucocorticoids were continued to be given, and the follow-up prognosis was good.

For such patients with mild to moderate ARDS, timely and appropriate respiratory support treatment should also be administered. Non-invasive positive pressure ventilation and high-flow nasal cannula oxygen therapy are non-invasive respiratory support methods that can avoid a series of serious complications caused by invasive operations. However, non-invasive positive pressure ventilation is often intolerable in clinical applications due to reasons such as facial pressure injuries, eating and drinking, expectoration, and communication limitations. In contrast, high-flow nasal cannula is easy to operate and manage, reduces the risk of infection, and can produce an effect similar to positive end-expiratory pressure to maintain alveolar opening. According to the Berlin Criteria, this case of nitrate fume inhalation resulted in mild to moderate ARDS. However, due to the patient’s intolerance to non-invasive positive pressure ventilation, we prioritized high-flow nasal cannula as the respiratory support modality. This treatment plan not only ensures comfort but also improves the patient’s oxygenation and relieves the asthma symptoms, achieving a good therapeutic effect.

In past clinical work, chest X-ray and CT are routinely used to evaluate pulmonary edema, but it cannot be ignored that bedside pulmonary ultrasound may be more convenient and accurate in identifying pulmonary edema [12–14]. In pulmonary ultrasound, if B-lines formed by the interface reflection of the coexistence of gas and liquid can be seen, pulmonary edema can be judged to a certain extent. Relevant data show that when three or more B-lines are seen between the bilateral intercostal spaces, the sensitivity and specificity of diagnosing pulmonary edema are much higher than those of physical examination and chest X-ray [15]. Picano et al. studied and believed that performing pulmonary ultrasound exploration on the anterior and lateral chest of the patient, the total number of B-lines can reflect the degree of pulmonary edema [2]. Based on the observation of this case, the patient’s condition continued to worsen in the first two days of the onset, and the lung images of the two CT examinations were similar, suggesting that the progress of the clinical symptoms is not completely synchronized with the CT changes. However, the number of B-lines seen by ultrasound increased. Therefore, in the dynamic evaluation of pulmonary edema, pulmonary ultrasound examination may be more sensitive to reflect the changes of the condition than CT examination, which is worthy of further verification and application in clinical practice. This case suggests that the use of pulmonary ultrasound technology not only enables a rapid diagnosis but also semi-quantitatively assesses the patient’s non-cardiogenic pulmonary edema, thereby enabling a faster evaluation of the patient’s condition changes and the formulation and adjustment of the treatment plan. For example, adjusting the dosage and usage of glucocorticoids, the selection of respiratory support methods, and the adjustment of respiratory parameters during the treatment process. Furthermore, adjustments to the treatment protocol should not be based solely on changes in bedside ultrasound findings, but rather require comprehensive evaluation incorporating additional laboratory tests and imaging studies [16].

## Conclusion

This case emphasizes that the occurrence of delayed pulmonary edema cannot be overlooked in the diagnosis and treatment process of patients inhaling nitric acid fumes, and the significance of enhancing bedside pulmonary ultrasound examination is put forward. Excellent bedside ultrasound skills can help clinicians identify and judge the degree of non-cardiogenic pulmonary edema more quickly and accurately, formulate and adjust reasonable treatment plans, so that patients can obtain better treatment effects and prognosis.

## Electronic supplementary material

Below is the link to the electronic supplementary material.

Supplementary Material 1

## Data Availability

Availability of data and materials：The data used and analyzed during the current study are available from the corresponding author in response to reasonable requests.
